# Genome-Wide Identification and Characterization of the MYB Transcription Factor Family in *Platycodon grandiflorus* and Its Potential Involvement in Flavonoid Biosynthesis Regulation

**DOI:** 10.3390/genes17060638

**Published:** 2026-05-30

**Authors:** Yalan Feng, Yeying Wu, Siyuan Ren, Zhonghao An, Xiaokang Gao, Xiaohua Wang, Na Shen, Chao Ma

**Affiliations:** 1 College of Life Sciences, Wuchang University of Technology, Wuhan 430200, China; yalanfeng@wut.edu.cn (Y.F.); colour0924@wut.edu.cn (Y.W.); rsy2024@wut.edu.cn (S.R.); azh2024@wut.edu.cn (Z.A.); 120162025@wut.edu.cn (X.G.); 120150028@wut.edu.cn (X.W.); 120161366@wut.edu.cn (N.S.); 2 Synergy Innovation Center of Biological Peptide Antidiabetics of Hubei Province, College of Life Science, Wuchang University of Technology, Wuhan 430200, China; 3 College of Agriculture, Henan University of Science and Technology, Luoyang 471023, China

**Keywords:** *Platycodon grandiflorus*, MYB transcription factor, genome-wide identification, flavonoid biosynthesis

## Abstract

**Background:** MYB transcription factors are key regulators of plant growth, development, secondary metabolism, and stress responses. However, this family has not been systematically characterized in the traditional medicinal plant *Platycodon grandiflorus*, and its roles in flavonoid biosynthesis remain largely unknown. **Methods:** We performed genome-wide identification of the MYB family using a combined HMMER and BLASTP approach with manual domain validation. Phylogenetic analysis was conducted on conserved MYB domains, followed by synteny, gene structure, conserved motif, and promoter cis-element analyses. Expression patterns under methyl jasmonate (MeJA) treatment were examined via transcriptomics and RT-qPCR. Protein-protein interaction networks were predicted using STRING based on *Arabidopsis* homologs. Subcellular localization of candidate proteins was tested in *Nicotiana benthamiana* leaf epidermal cells. **Results:** A total of 170 *PgMYB* members were identified, comprising 52.9% 1R-MYB and 44.1% 2R-MYB. They clustered into 26 subgroups (P1–P26), with 1R-MYBs enriched in subgroup P1 (82 members). Synteny analysis revealed 192 collinear blocks between *P. grandiflorus* and *Arabidopsis*, and all 26 syntenic gene pairs examined had Ka/Ks < 1, indicating strong purifying selection. Promoter regions were enriched in hormone- (72.9% ABA-responsive) and stress-responsive elements. Nine selected genes showed consistent MeJA-induced expression changes between RNA-seq and RT-qPCR. Integrated analysis of phylogeny, expression correlation, and predicted protein-protein interactions nominated *PgMYB47*, *PgMYB142*, and *PgMYB151* as candidate regulators of flavonoid biosynthesis. All three proteins localized to the nucleus in *N. benthamiana* cells. **Conclusions:** This study provides the first comprehensive characterization of the *P. grandiflorus* MYB family, highlighting its evolutionary conservation and expression dynamics. The nominated candidates offer a foundation for future functional validation of flavonoid biosynthesis regulation.

## 1. Introduction

The MYB (myeloblastosis) transcription factor family represents one of the largest families of transcription factors in eukaryotes and is widely distributed across higher plant genomes [1]. Members of this family contain a highly conserved DNA-binding domain at their N-terminus, typically consisting of 1–4 imperfect repeats, and can be divided into several subfamilies, including 1R-MYB, R2R3-MYB, 3R-MYB, and 4R-MYB, based on the number of repeats [2,3]. Among these, the R2R3-MYB subfamily is the most abundant and functionally diverse in plants, and is extensively involved in regulating key biological processes such as growth and development, secondary metabolism, and stress responses [4]. Notably, in the plant phenylpropanoid pathway, R2R3-MYB transcription factors often form a ternary MBW complex with bHLH and WD40 proteins to precisely regulate the expression of downstream biosynthetic genes for flavonoids, anthocyanins, thereby influencing plant color, flavor, and resistance [5,6]. Furthermore, plant hormone signals such as MeJA have been shown to regulate the accumulation of secondary metabolites by activating specific MYB transcription factors [7,8]. Currently, the MYB family has been fully characterized in the genomes of various plants, such as *Arabidopsis thaliana* [1] and rice [9], and research on its regulatory network has provided important genetic resources for the genetic improvement of crop quality and resistance.

*Platycodon grandiflorus* is a traditional medicinal and edible plant in China. Its dried roots are rich in various active ingredients such as triterpenoid saponins, polysaccharides, and flavonoids, and exhibit multiple pharmacological activities including expectorant, immunomodulatory, and antioxidant effects [10,11]. Among these, flavonoids, as one of the important active components of *P. grandiflorus*, show significant efficacy in anti-inflammatory, anticancer, and cardiocerebrovascular protection, and their biosynthesis and accumulation levels directly affect the quality of the medicinal material [12,13]. However, current research on the active ingredients of *P. grandiflorus* has mostly focused on saponins. The flavonoid biosynthetic pathway, particularly the key transcriptional regulatory mechanisms upstream of this pathway, has not been systematically resolved. As the core transcription factors regulating flavonoid synthesis, the composition, evolutionary characteristics, and regulatory functions of the MYB family in *P. grandiflorus* remain largely unknown, with only one R2R3-MYB repressor (*PlgMYBR1*) having been functionally implicated in anthocyanin biosynthesis to date [14]. This knowledge gap severely limits an in-depth understanding of the secondary metabolic regulatory network in *P. grandiflorus* and hinders genetic improvement efforts aimed at increasing flavonoid content through molecular breeding. Kim et al. [14] identified *PlgMYBR1* as a negative regulator of anthocyanin accumulation in *P. grandiflorus*, suggesting that MYB-mediated transcriptional repression also operates in this species. However, the broader regulatory network governing flavonoid biosynthesis remains to be elucidated. Our genome-wide identification thus complements this functional report and provides a resource for exploring the remaining 169 MYB members.

In recent years, the completion of the whole-genome sequencing of *P. grandiflorus* has provided a critical foundation for systematically identifying the MYB transcription factor family at the genome-wide level and exploring its functions [15]. This study represents the first genome-wide identification and bioinformatics analysis of the MYB transcription factor family based on the *P. grandiflorus* genome, including phylogenetic analysis, gene structure, conserved motifs, chromosomal distribution, synteny, and promoter cis-element analysis. Furthermore, by analyzing expression patterns across different tissues and in response to MeJA treatment, and by integrating protein-protein interaction (PPI) network and co-expression analyses, we aim to screen core candidate genes potentially involved in regulating flavonoid biosynthesis and to perform preliminary functional validation, such as subcellular localization. The objectives of this study were to comprehensively characterize the members and evolutionary patterns of the *P. grandiflorus* MYB transcription factor family, provide initial insights into its potential role in regulating flavonoid synthesis, and offer core genetic resources and a theoretical basis for elucidating the transcriptional regulatory network governing the biosynthesis of medicinal active ingredients in *P. grandiflorus*. Ultimately, these findings are expected to support molecular design breeding and quality-oriented improvement of *P. grandiflorus*.

## 2. Materials and Methods

### 2.1. Identification of MYB Transcription Factor Family Members in P. grandiflorus

The genome sequence, amino acid sequences, and gene annotation file (GFF) of *P. grandiflorus* were downloaded from the National Genomics Data Center (https://ngdc.cncb.ac.cn/) under accession number GWHARYT00000000. To ensure comprehensive and unbiased identification of all MYB family members (including divergent 1R, 2R, 3R, and 4R types), a two-pronged search strategy was adopted. First, a hidden Markov model (HMM)-based search was performed using the MYB domain profile (PF00249) obtained from the Pfam database (http://pfam.xfam.org/). Specifically, the HMMER 3.0 software (hmmsearch) was run against the *P. grandiflorus* proteome with an E-value threshold of 1 × 10^−5^. Second, a complementary BLASTP search was conducted using all MYB protein sequences from *Arabidopsis thaliana* (including 126 R2R3-MYBs and all known 1R, 3R, and 4R MYBs) as queries, with an E-value cutoff of 1 × 10^−5^. The candidate sequences from both approaches were merged, and redundant entries were removed. Each candidate was then subjected to manual validation using the NCBI Batch CD-Search tool (https://www.ncbi.nlm.nih.gov/Structure/bwrpsb/bwrpsb.cgi) (accessed on 15 April 2025) and the SMART database (http://smart.embl-heidelberg.de/, accessed on 15 April 2025). Proteins lacking a complete MYB domain (i.e., missing the conserved tryptophan residues or containing truncated repeats) were discarded. In addition, sequences with obvious fragmentation or artificially fused domains were excluded. For the final retained members, the type and number of MYB repeats (1R, 2R, 3R, or 4R) were determined based on domain architecture. This rigorous pipeline ensures high confidence in both the membership and classification of the PgMYB family.

### 2.2. Phylogenetic Tree Construction and Subgroup Classification

To obtain a robust phylogenetic framework for functional inference, we reconstructed the phylogeny using only the conserved MYB DNA-binding domain (typically 90–120 amino acids covering the R repeats). The MYB domain sequences of 170 PgMYB proteins and 126 *Arabidopsis* R2R3-MYB proteins (plus representative 1R, 3R, 4R MYBs) were extracted based on Pfam annotation (PF00249). Multiple sequence alignments of *P. grandiflorus* MYB and *Arabidopsis thaliana* R2R3-MYB protein sequences were performed using MEGA11 software (Version 11.0.13; Mega Software, Philadelphia, PA, USA) with the Neighbor-Joining (NJ) method. A phylogenetic tree was constructed using the P-distance model with bootstrap values calculated from 1000 replicates. Physicochemical properties, including amino acid count, molecular weight, and instability index, were computed using the ExPASy ProtParam tool (https://web.expasy.org/protparam/, accessed on 15 April 2025) (Version 2.14.0; National Center for Biotechnology Information, Bethesda, MD, USA). To validate the robustness of the NJ topology, we also performed a maximum likelihood (ML) analysis (see Supplementary Figure S1 and Supplementary Methods for details).

### 2.3. Chromosomal Localization, Synteny Analysis, and Selection Pressure Analysis

TBtools software (Version 2.481; South China Agricultural University, Guangzhou, China) was used to draw the chromosomal localization map of *P. grandiflorus* MYB genes, and they were numbered according to their chromosomal positions. Synteny analysis was performed using the MCScanX module with an E-value threshold of 1 × 10^−5^, and the syntenic relationships were visualized using the Advanced Circos function in TBtools. For each identified syntenic gene pairs, the nonsynonymous substitution rate (Ka), synonymous substitution rate (Ks), and their ratio (Ka/Ks) were calculated using TBtools software.

### 2.4. Conserved Motif and Gene Structure Analysis

Using the *P. grandiflorus* genome gff file, the gene structures (exon-intron organization) of MYB family members were visualized with TBtools software. Conserved motifs in *P. grandiflorus* MYB proteins were predicted using the MEME suite (https://meme-suite.org/meme/tools/meme) (accessed on 9 May 2025) [16] with a maximum of 20 motifs and default parameters for all other settings. Visualization was performed using TBtools [17].

### 2.5. Promoter cis-Element Prediction

The 2000 bp DNA sequence upstream of the start codon (ATG) of each *P. grandiflorus* MYB family member was extracted and submitted to the PlantCARE database (http://bioinformatics.psb.ugent.be/webtools/plantcare/html/) (accessed on 15 April 2025) for promoter cis-element prediction. Candidate binding elements were screened, and the results were visualized using TBtools software.

### 2.6. Gene Expression Pattern Analysis

Transcriptome datasets of twelve *P. grandiflorus* whole-roots were obtained from the NCBI database (accession numbers: SRX22242566, SRX22242563, SRX22242562, SRX22242569, SRX22242568, SRX22242567, SRX22242572, SRX22242571, SRX22242570, SRX22242573, SRX22242565, SRX22242564), corresponding to samples treated with 100 μmol·L^−1^ MeJA for different time points. After log_2_(FPKM+1) transformation of the FPKM values, a heatmap of *P. grandiflorus* MYB gene expression was drawn using TBtools software.

### 2.7. Protein-Protein Interaction Network Prediction and Expression Correlation Analysis

Based on the expression patterns of flavonoid biosynthesis-related structural genes in *P. grandiflorus* whole-roots under different treatments, the Pearson correlation coefficients between the expression levels of these structural genes and those of *P. grandiflorus* MYB genes were calculated, and the results were visualized as a heatmap using TBtools software. A predicted protein-protein interaction (PPI) network was constructed using the STRING database (https://www.string-db.org/) with the highest confidence score (0.900). Because no direct interaction data are available for *P. grandiflorus*, the network was inferred by transferring experimentally validated or predicted interactions from *Arabidopsis thaliana* homologs. Specifically, for each PgMYB and each flavonoid biosynthetic enzyme, the corresponding Arabidopsis homologs were identified by reciprocal BLAST (Version 2.14.0; National Center for Biotechnology Information, Bethesda, MD, USA), and their interactions were retrieved from STRING. This approach provides a hypothesis-generating framework but does not demonstrate direct or cell-type-specific interactions in *P. grandiflorus*.

### 2.8. Plant Material, Hormone Treatment, and RT-qPCR Validation

*P. grandiflorus* plants used in this study were grown in the pot culture base of Wuchang University of Technology. After 90 days of growth, plants were treated with 100 μmol·L^−1^ each of indole-3-acetic acid (IAA), gibberellic acid (GA), abscisic acid (ABA), and MeJA, respectively, while normally growing plants served as controls. Three independent biological replicates were performed for each treatment and the control group. Each replicate consisted of roots pooled from three individual plants. After 12 h of treatment, whole-roots from each group were collected and immediately frozen in liquid nitrogen for storage. The plants used for RT-qPCR were grown and treated separately from those used for RNA-seq. Total RNA was extracted from these root samples using TRIzol reagent (TaKaRa, 9108Q, manufactured by Baosheng Bioengineering (Dalian) Co., Ltd., Dalian, China), and cDNA was synthesized by reverse transcription (TaKaRa, RR047Q, manufactured by Baosheng Bioengineering (Dalian) Co., Ltd., Dalian, China) for real-time quantitative PCR (RT-qPCR) analysis. The RT-qPCR reaction system and procedure followed the instructions of the ChamQ Universal SYBR qPCR Master Mix kit (Vazyme, Q221, Vazyme Biotech Co., Ltd., Nanjing, China). The relative expression levels were calculated using the 2^−ΔΔCt^ method [18]. Nine *PgMYB* genes identified from the transcriptome sequencing were selected based on the following criteria: (i) |log_2_(fold change)| > 1 in at least one time point under MeJA treatment in the RNA-seq data; (ii) FPKM > 5 in at least one sample; and (iii) representatives of different response patterns (up-regulated, down-regulated, and unchanged). These included *PgMYB14*, *PgMYB15*, *PgMYB47*, *PgMYB62*, *PgMYB99*, *PgMYB111*, *PgMYB142*, *PgMYB150*, and *PgMYB167*. The primer sequences are listed in Table 1, with β-actin as the internal reference gene. The concentration of 100 μmol·L^−1^ was selected based on preliminary experiments and previous studies in other medicinal plants, such as *Glycyrrhiza inflata* [19] and *Ginkgo biloba* [20], in which this concentration effectively induced the expression of secondary metabolism-related MYB genes without causing visible growth inhibition. A full dose-response (hormetic) window was not established in this study; therefore, the 12 h time point may capture both early and adaptive transcriptional responses.

### 2.9. Subcellular Localization of Candidate Genes in N. benthamiana Leaf Epidermal Cells

To verify the subcellular localization of PgMYB47, PgMYB142, and PgMYB151, methods described by Wang et al. [21] were followed. The coding sequences of the three genes without the stop codon were amplified and cloned into the super1300-GFP-N vector to generate the transient expression constructs 35S::PgMYB47-GFP, 35S::PgMYB142-GFP, and 35S::PgMYB151-GFP. *Agrobacterium* cells containing the recombinant vectors were cultured to an OD_600_ of approximately 0.8, harvested by centrifugation, resuspended to the same OD_600_, and infiltrated into the epidermal cells of *N. benthamiana* leaves. Nuclei were counterstained with DAPI (4′,6-diamidino-2-phenylindole). Co-localization of GFP fluorescence with DAPI signal was used to determine nuclear localization. The green fluorescence signals of the fusion proteins were observed under a confocal microscope. The cDNA used was derived from reverse transcription of *P. grandiflorus* whole-root RNA, and the sequence information for *PgMYB47*, *PgMYB142*, and *PgMYB151* came from the genome data in Section 2.1.

## 3. Results

### 3.1. Identification and Phylogenetic Analysis of the MYB Gene Family in P. grandiflorus

Using *Arabidopsis* MYB protein sequences as a reference, a combination of BLASTP alignment and HMM searches, together with NCBI Batch CD-Search for conserved domain validation of candidate genes, ultimately identified 170 MYB transcription factor family members containing complete MYB domains in the *P. grandiflorus* genome. Based on the type and number of R domains, they were divided into four subfamilies: 1R-MYB had the most members (90, accounting for 52.9% of the total); 2R-MYB had 75 members (44.1%); 3R-MYB had 4 members (2.4%); and 4R-MYB had 1 member (0.6%). The members were named PgMYB1 to PgMYB170 according to their positions on the chromosomes. The dual-strategy search (HMMER + BLASTP) combined with manual domain validation ensured that even highly divergent 1R and 3R/4R members were captured and that partial or redundant sequences were excluded (see Section 2.1 for details).

**Figure 1:**
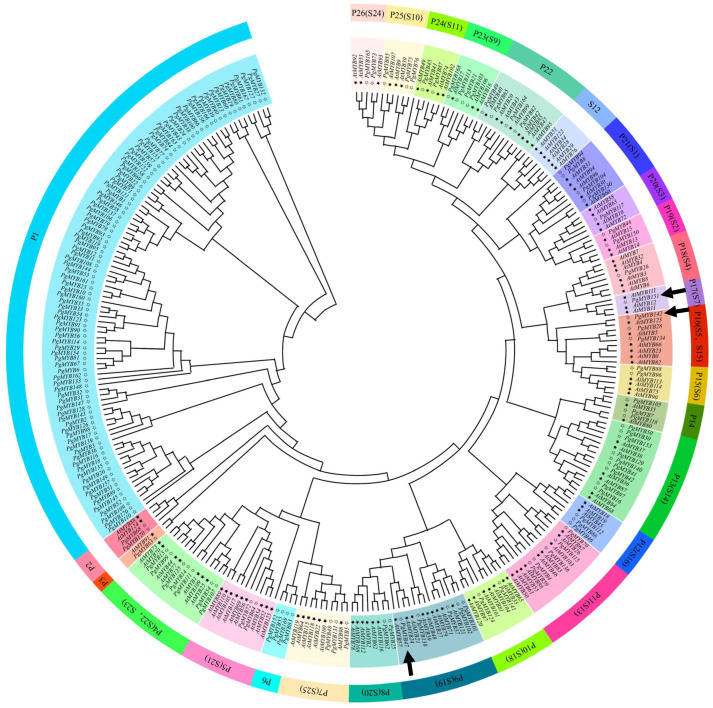
Phylogenetic tree of MYB gene family members from *Platycodon grandiflorus* (☆) and *Arabidopsis thaliana* (★). The tree was constructed using the conserved MYB DNA-binding domain (PF00249) of 170 PgMYB and 126 Arabidopsis R2R3-MYB proteins (plus representative 1R, 3R, and 4R MYBs). Multiple sequence alignment was performed with MEGA11 using the Neighbor-Joining (NJ) method under the P-distance model, and branch support was assessed by 1000 bootstrap replicates. Bootstrap values (≥50%) are shown at nodes. The 170 PgMYB members were classified into 26 subgroups (P1–P26) based on clustering with Arabidopsis MYBs of known subfamilies. Subgroup P1 contains predominantly 1R-MYBs (82 PgMYB members). Subgroup P6 consists exclusively of four PgMYB 3R-type proteins with no *Arabidopsis* counterpart. The single 4R-MYB (PgMYB63) clusters with AtMYB91 in subgroup P3. Key candidate genes PgMYB47, PgMYB142, and PgMYB151 are highlighted with bold arrows. Branches are colored by MYB type: 1R (green), 2R (blue), 3R (orange), and 4R (purple). The scale bar represents the number of substitutions per site.

The 170 PgMYB members were distributed across 26 subgroups (P1–P26) (Figure 1), which were defined by clustering with *Arabidopsis* MYBs of known subfamilies. Consistent with previous classification, 1R-MYB members were largely restricted to subgroup P1, which contained 92 members (82 from *P. grandiflorus*). Subgroup P6 consisted exclusively of four PgMYB 3R-type proteins, with no *Arabidopsis* counterpart, suggesting a lineage-specific expansion. The single 4R-MYB (PgMYB63) clustered with AtMYB91 in subgroup P3. Notably, 24 out of 26 subgroups contained both *P. grandiflorus* and *Arabidopsis* MYB members, predominantly of the 2R-MYB type. However, no PgMYB member was detected in the *Arabidopsis* S12 subgroup, indicating possible lineage-specific loss or divergence. The NJ topology was independently validated by a ML analysis based on the same conserved domain alignment, which produced highly congruent clustering patterns (Supplementary Figure S1).

### 3.2. Physicochemical Properties and Subcellular Localization Analysis of P. grandiflorus MYB Family Proteins

The physicochemical properties of the 170 *P. grandiflorus* MYB proteins were predicted using TBtools software (Table S1). The amino acid sequence lengths ranged from 78 to 1948 aa, and molecular weights ranged from 8.67 to 210.38 kDa. The theoretical isoelectric points (pI) ranged from 4.42 to 9.89, with 57.6% of the proteins being acidic (pI < 7) and 42.4% basic. The protein instability index ranged from 31.41 to 82.21, with only 11 proteins (6.5%) having an instability index less than 40, classified as stable proteins. The aliphatic index ranged from 45.11 to 85.37, and the grand average of hydropathicity (GRAVY) ranged from −1.442 to −0.174, all negative, indicating that all PgMYB proteins are all predicted to be hydrophilic. Subcellular localization prediction results (Supplementary Table S1) showed that the 170 PgMYB proteins were mainly distributed in four subcellular compartments: the nucleus (90.0%), chloroplast (4.7%), cytoplasm (3.5%), and peroxisome (1.7%). Thus, the vast majority of *P. grandiflorus* MYB proteins are predicted to localize to the nucleus in *N. benthamiana* leaf epidermal cells, which is consistent with their expected function as transcription factors.

### 3.3. Chromosomal Localization and Synteny Analysis of the MYB Gene Family in P. grandiflorus

**Figure 2:**
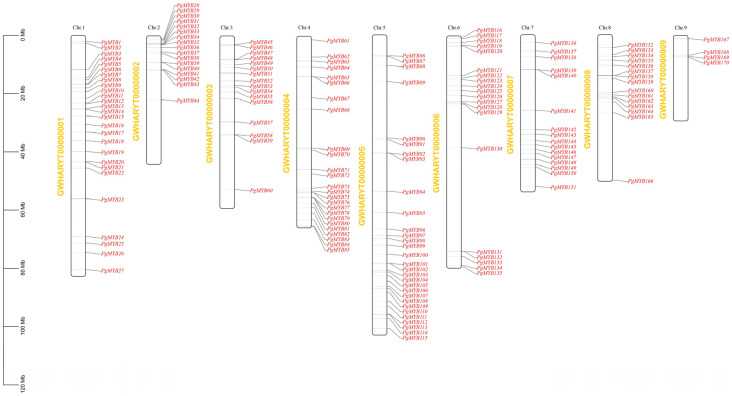
Chromosomal mapping of MYB gene in *P. grandiflorus*.

Chromosomal localization analysis (Figure 2) showed that the 170 *P. grandiflorus* MYB genes were unevenly distributed across 9 chromosomes, with most genes clustering on the chromosomes. Chromosome 5 (Chr.5) harbored the highest number of genes (30, 17.6% of the total), while Chromosome 9 (Chr.9) had the fewest (4, 2.4%).

**Figure 3:**
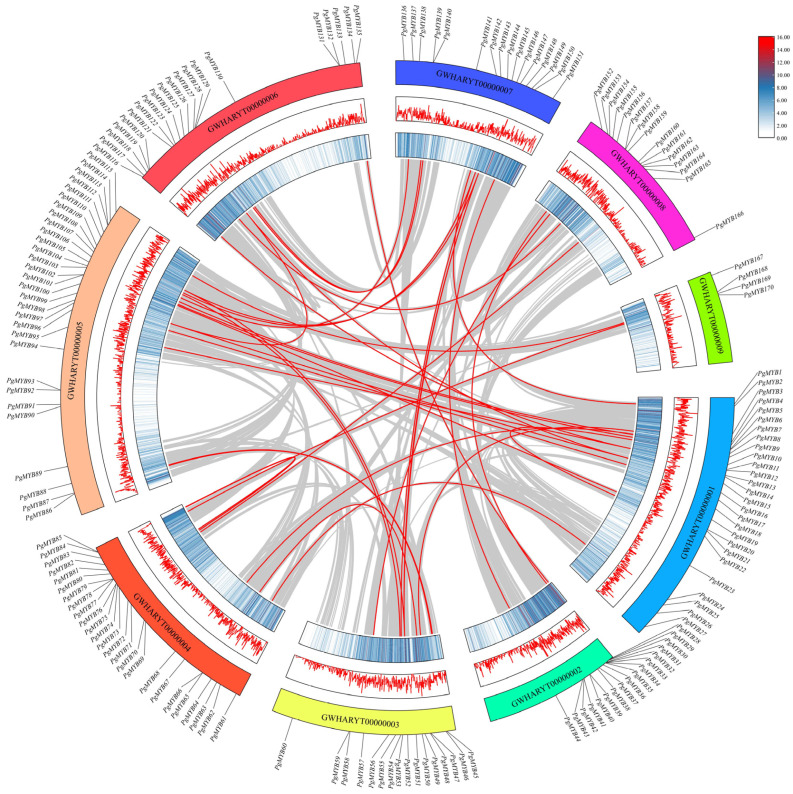
Collinearity analysis of PgMYB genes in *P. grandiflorus*. Circular plot showing collinearity among PgMYB genes. Red lines: synteny between PgMYB genes; Gray lines: all genomic collinearity events. The scale represents gene density, where red corresponds to high density and white to low density.

**Figure 4:**
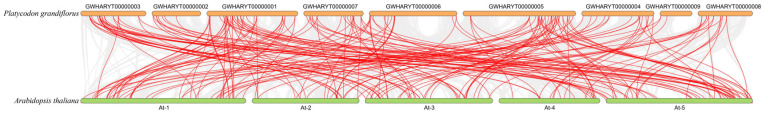
Interspecific collinearity between MYB genes in *P. grandiflorus* and *Arabidopsis thaliana*. Red lines denote syntenic events between *P. grandiflorus* and *Arabidopsis thaliana* MYB genes, while gray lines represent all collinear events.

Synteny analysis results (Figure 3) revealed 1763 syntenic regions within the *P. grandiflorus* genome, of which 36 involved the MYB transcription factor family members. To further investigate the evolutionary constraints on MYB syntenic gene pairs, the Ka/Ks ratios of 26 syntenic gene pairs were calculated (Supplementary Table S2). The Ka/Ks values for all gene pairs ranged from 0.038999359 to 0.337566796, all substantially less than 1, indicating that these *PgMYB* genes have undergone purifying selection during evolution. Interspecific synteny analysis between *P. grandiflorus* and *Arabidopsis* (Figure 4) identified a total of 192 syntenic regions between the two species, involving 106 *P. grandiflorus* MYB genes and 161 *Arabidopsis* MYB genes. This result suggests that these MYB members are highly conserved evolutionarily and have been retained and duplicated.

### 3.4. Conserved Motif and Gene Structure Analysis of P. grandiflorus MYB Transcription Factor Family Members

**Figure 5:**
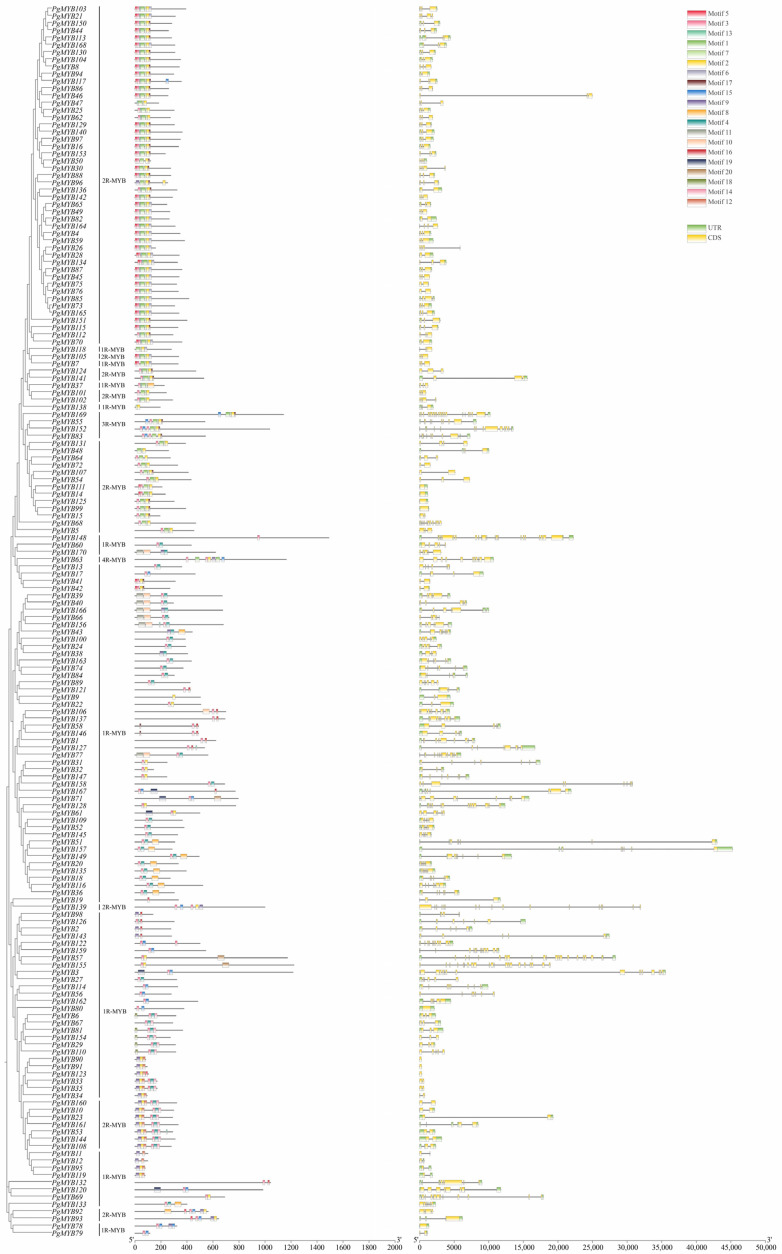
Conserved motif and gene structure of MYB gene in *P. grandiflorus*.

Conserved motif prediction was performed on the 170 PgMYB proteins using the MEME online tool, and 20 conserved motifs were identified (Figure 5). The number of motifs per PgMYB family member ranged from 1 to 8, with variations in both the number and distribution positions of motifs among different members. Further analysis revealed that, except for PgMYB47, PgMYB48, and PgMYB96, all other 2R-MYB type members contained both Motif2 and Motif3; 1R-MYB type members contained either Motif2 or Motif3, and some members contained both motifs; 3R-MYB and 4R-MYB type members all contained Motif15 in addition to Motif2 and Motif3. These results indicate that Motif2, Motif3, and Motif15 are signature conserved motifs of the PgMYB family.

Gene structure analysis (Figure 5) showed that the number of exons in all PgMYB members ranged from 1 to 21, and the number of introns ranged from 0 to 22. Among them, nine genes contained no introns; genes with three exons were the most abundant (53 genes, 31.2%); 47 genes (27.6%) lacked untranslated regions (UTRs). PgMYB148 had the highest number of introns (22), and PgMYB155 had the highest number of exons (21). The observed differences in exon and intron numbers and positions among PgMYB genes may constitute the structural basis for their diverse regulatory functions.

### 3.5. Promoter cis-Element Analysis of the P. grandiflorus MYB Gene Family

**Figure 6:**
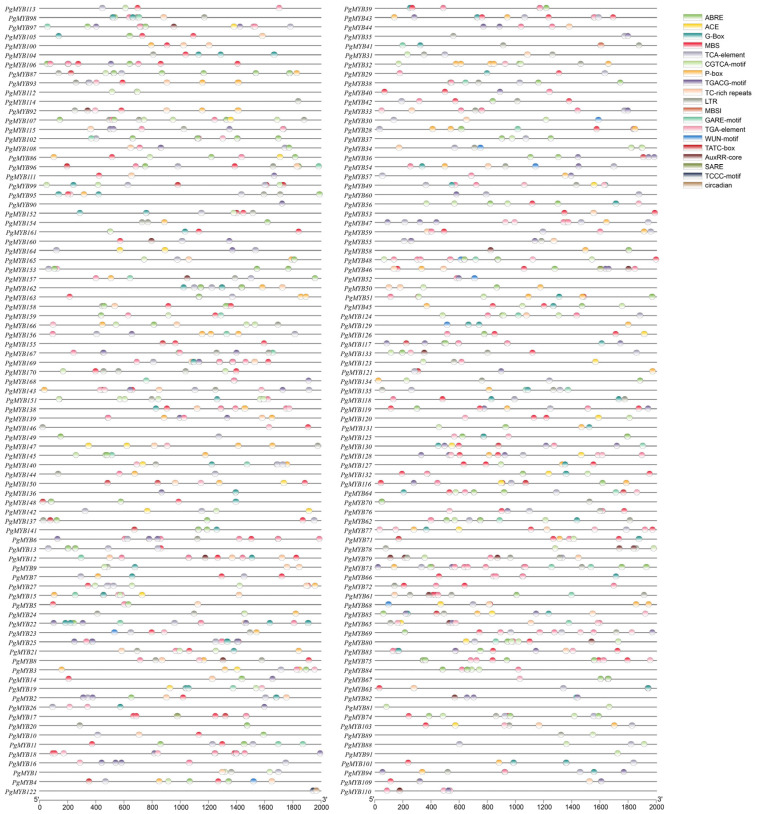
Cis-acting element of the promoter of the MYB gene in *P. grandiflorus*.

To explore the potential transcriptional regulatory functions of *PgMYB* genes, cis-elements located within the 2000 bp promoter region upstream of the start codon of each member were predicted. A total of 19 types of cis-elements were identified, which could be classified into three categories: hormone response, growth and development regulation, and stress response (Figure 6). These included ten types of hormone-related elements, five types of growth- and development-related elements, and four types of stress-response elements. Examples of specific elements included light-responsive elements, drought-inducible elements, defense and stress elements, low-temperature response elements (LTR), gibberellin-responsive, and auxin-responsive.

Among the hormone-related elements, the ABA response element was the most widely distributed, present in 124 genes (72.9%), whereas the auxin response element had the lowest frequency, occurring in only 58 genes (34.1%). Among the stress-related elements, the drought response element was the most common, found in 83 genes (48.8%), while the wound response element had the lowest frequency, present in only 5.3%. In addition, 5.9% of *PgMYB* gene promoter regions contained cis-elements related to flavonoid biosynthesis. Further analysis revealed that PgMYB73 contained the greatest variety of cis-elements, whereas PgMYB114 contained only one LTR, making it the member with the fewest elements.

### 3.6. Tissue Expression Patterns of the P. grandiflorus MYB Gene Family

**Figure 7:**
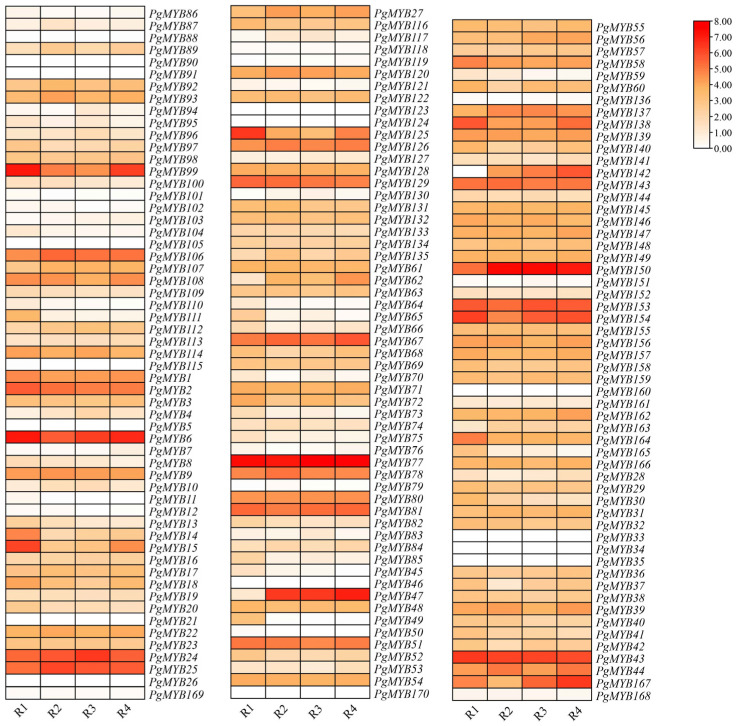
Expression patterns of *PgMYB* genes in *P. grandiflorus* whole-roots at 0 h (R1, control), 12 h (R2), 24 h (R3), and 48 h (R4) after MeJA treatment. The color scale represents log_2_(FPKM+1) values.

Based on transcriptome data, the expression changes of 170 *PgMYB* genes in *P. grandiflorus* whole-roots at different time points after treatment with 100 μmol·L^−1^ MeJA (R1: control, 0 h; R2: 12 h treatment; R3: 24 h treatment; R4: 48 h treatment) were analyzed. A heatmap (Figure 7) was generated after log_2_(FPKM+1) transformation of the FPKM values. The results showed that 56 *PgMYB* members (32.9%) exhibited relatively high expression (FPKM > 10) in the control (R1). After MeJA treatment, 12 members (7.1%) were significantly up-regulated (expression fold change > 2) at all three time points (R2, R3, and R4). Among these, *PgMYB142* was barely detectable in the control, its expression was activated by MeJA treatment, and its expression level gradually increased with treatment time. *PgMYB47* showed the largest increase following MeJA treatment, with an expression level 101-fold higher than in the control at the R3 time point. Conversely, 30 *PgMYB* members (17.6%) were significantly down-regulated (fold change < −2) at the R2 time point. Notably, the expression of *PgMYB167* decreased first and then increased over the treatment period, and its relative expression level at the R4 time point was significantly higher than that in the control. The expression levels described above were measured from whole-root homogenates and therefore represent average transcript abundance across all root cell types.

### 3.7. Predicted Protein-Protein Interaction Network Between PgMYBs and Flavonoid Biosynthetic Enzymes Based on Arabidopsis Homologs

**Figure 8:**
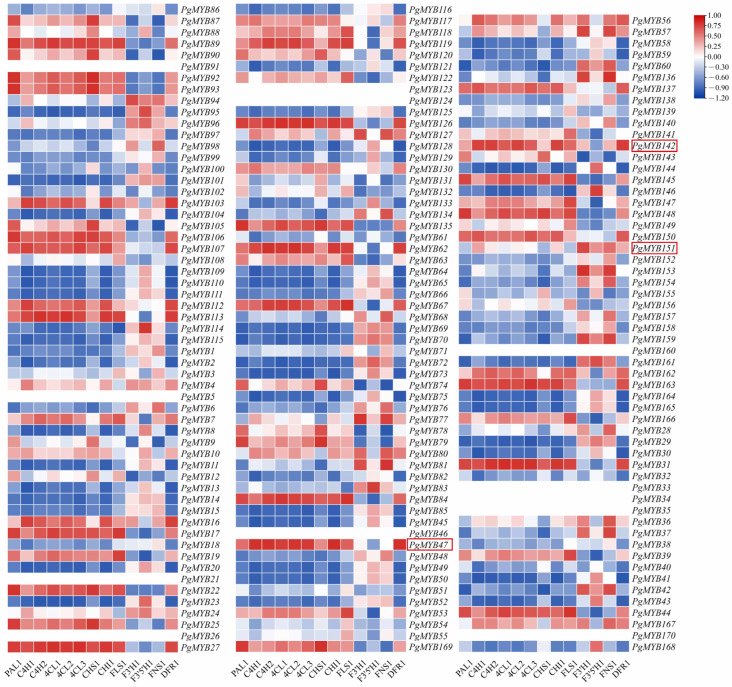
Heatmap of Pearson correlation coefficients (r) between the expression levels of *PgMYB* genes and flavonoid biosynthetic structural genes in *P. grandiflorus* roots under MeJA treatment (R1–R4). The color scale ranges from blue (r = −1, negative correlation) to red (r = +1, positive correlation). Darker shades indicate higher correlation coefficients; red represents positive correlations, and blue represents negative correlations. Structural gene abbreviations: PAL, phenylalanine ammonia-lyase; C4H, cinnamate-4-hydroxylase; 4CL, 4-coumaroyl-CoA ligase; CHS, chalcone synthase; CHI, chalcone isomerase; FNS, flavone synthase; FLS, flavonol synthase; F3′H, flavonoid 3′-hydroxylase; F3′5′H, flavonoid 3′,5′-hydroxylase; DFR, dihydroflavonol reductase. Candidate MYB genes (*PgMYB47*, *PgMYB142*, and *PgMYB151*) are indicated by red boxes. The expression data were log_2_(FPKM+1) transformed before correlation calculation.

Based on transcriptome data, ten types of structural genes involved in flavonoid biosynthesis were screened, including phenylalanine ammonia-lyase (PAL), chalcone isomerase (CHI), cinnamate-4-hydroxylase (C4H), 4-coumaroyl-CoA ligase (4CL), chalcone synthase (CHS), flavone synthase (FNS), flavonol synthase (FLS), flavonoid 3′-hydroxylase (F3′H), flavonoid 3′,5′-hydroxylase (F3′5′H), and dihydroflavonol reductase (DFR). A total of 13 transcripts (including multiple gene family members) of these structural genes were obtained from *P. grandiflorus* whole-roots under different treatments. Based on the expression patterns of these transcripts and *PgMYB* genes, Pearson correlation coefficients were calculated, and a correlation heatmap was generated using TBtools software (Figure 8).

The results showed that 12 *PgMYB* members had no significant correlation with the screened flavonoid synthesis structural genes (|r| < 0.5). Among the 13 structural gene transcripts, *C4H1* and *DFR1* were the two genes that showed the highest number of highly positive or negative correlations (|r| > 0.9) with *PgMYB* members, each significantly correlated with 38 *PgMYB* members. *F3′H1* showed the fewest correlations, with only eight. Further analysis revealed that *PgMYB47* was highly positively correlated (r > 0.9) with seven structural gene transcripts, involving four types of structural genes: *C4H*, *4CL*, *CHI*, and *DFR*. *PgMYB14* showed a high negative correlation (r < −0.8) with eight structural gene transcripts, involving five types: *PAL*, *C4H*, *4CL*, *CHI*, and *DFR*.

**Figure 9:**
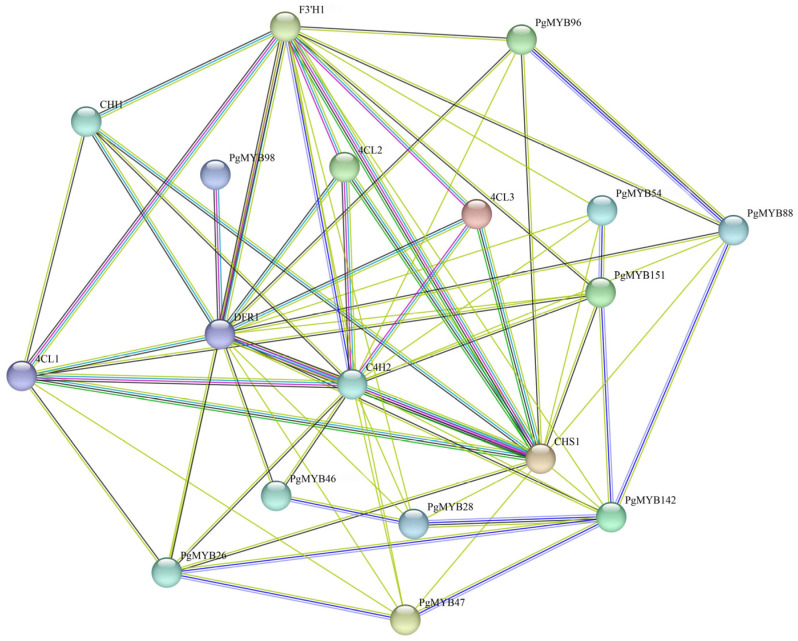
Predicted protein-protein interaction network between PgMYB proteins and flavonoid biosynthetic enzymes. Interactions were inferred from *Arabidopsis thaliana* homologs using the STRING database (confidence score ≥ 0.900). This prediction does not demonstrate direct or cell-type-specific interactions in *P. grandiflorus*. Edges represent protein-protein associations. Line colors indicate the type of evidence supporting the interaction: Turquoise (from curated databases), Purple (experimentally determined), Green (gene neighborhood), Red (gene fusions), Blue (gene co-occurrence), Light yellow (textmining), Black (co-expression), and Light purple (protein homology).

To screen for *PgMYB* genes potentially involved in regulating flavonoid synthesis, a predicted PPI network was constructed based on *Arabidopsis* homologous protein data. Interaction analysis was performed between PgMYB proteins and the 13 flavonoid biosynthesis structural proteins described above, retaining only protein pairs with predicted interactions. The rsulting network (Figure 9) suggested that, based on their *Arabidopsis* homologs using the STRING database, ten PgMYB proteins potentially interact with eight flavonoid biosynthetic enzymes. These interactions are predictions and do not represent experimentally validated PPIs in *P. grandiflorus*. Furthermore, transcription factors typically regulate metabolic pathways by binding to promoter regions of target genes rather than by directly interacting with biosynthetic enzymes. For example, PgMYB47 was predicted to interact with C4H2, 4CL1, CHS1, and DFR1. However, these are in silico predictions that require experimental validation (e.g., by yeast two-hybrid or co-immunoprecipitation), and do not provide information on cell-type specificity.

### 3.8. Expression Analysis of P. grandiflorus MYB Genes Under Different Exogenous Hormone Treatments

**Figure 10:**
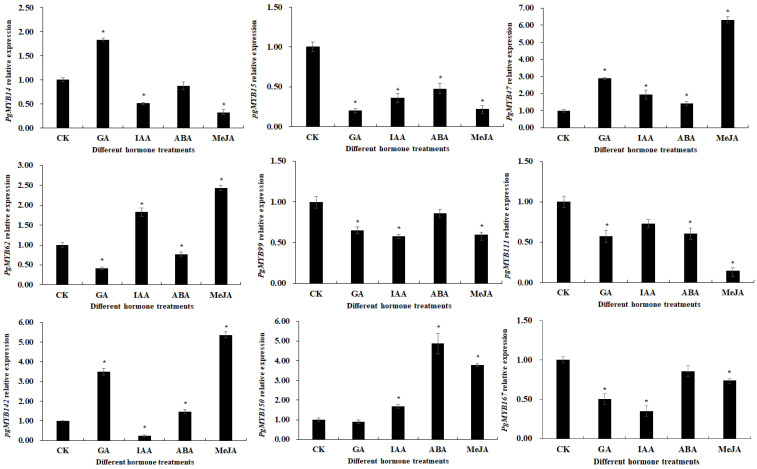
Expression analysis of nine selected *PgMYB* genes in *P. grandiflorus* whole-roots after treatment with 100 μmol·L^−1^ IAA, GA, ABA, or MeJA for 12 h, measured by RT-qPCR. Asterisks indicate significant differences (*p* < 0.05) compared with the untreated control.

To explore the response of *PgMYB* genes to different exogenous hormones, nine representative genes were selected for RT-qPCR analysis. The results (Figure 10) showed that under four hormone treatments (IAA, GA, ABA, and MeJA), each *PgMYB* gene exhibited a distinct expression pattern, with relative expression levels showing either up-regulation or down-regulation. Among them, the expression of *PgMYB15*, *PgMYB99*, *PgMYB111*, and *PgMYB167* was down-regulated after all four hormone treatments. In contrast, *PgMYB47* was significantly up-regulated after all four hormone treatments. Further analysis showed that under GA treatment, *PgMYB142* exhibited the largest increase, with a relative expression level 3.5-fold higher than that of the control. Under MeJA treatment, the up-regulation of *PgMYB47* expression was the most pronouced, reaching 6.3-fold increase, the highest among the nine tested genes. Additionally, under MeJA treatment, the expression patterns of the nine genes were consistent with the transcriptome sequencing results (Figure 7), validating the reliability of the RNA-Seq data. It should be emphasized that all expression correlations and predicted interactions presented here are based on transcriptomic data and homology inference, not on direct measurements of flavonoid accumulation. Thus, these results are hypothesis-generating and require functional validation.

### 3.9. Subcellular Localization of PgMYB47, PgMYB142, and PgMYB151 in N. benthamiana Leaf Epidermal Cells

**Figure 11:**
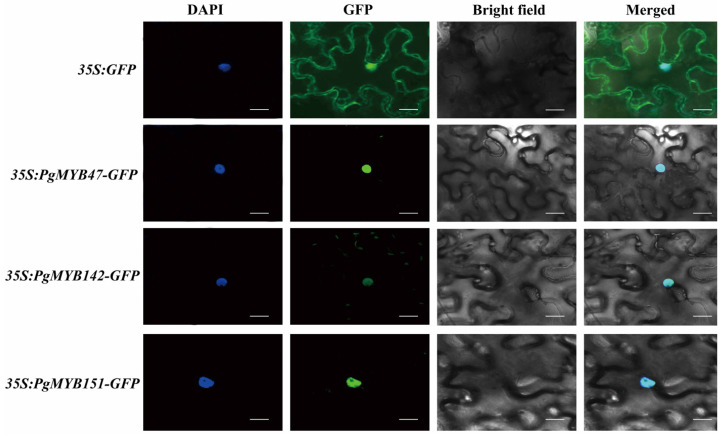
Subcellular localization of *PgMYB47*, *PgMYB142*, and *PgMYB151* in *N. benthamiana* leaf epidermal pavement cells. DAPI nuclear stain (blue), GFP fluorescence (green), and merged images (GFP + DAPI + bright field) are shown. Scale bar = 20 μm.

To determine the subcellular localization of PgMYB47, PgMYB142, and PgMYB151, the 35S::PgMYB47-GFP, 35S::PgMYB142-GFP, and 35S::PgMYB151-GFP fusion expression vectors were constructed and transiently expressed in *N. benthamiana* leaf epidermal cells. Confocal microscopy observation results (Figure 11) showed that the empty vector 35S::GFP produced green fluorescence signals in both the plasma membrane and nucleus of *N. benthamiana* leaf epidermal cells. In contrast, the green fluorescence signals from the fusion proteins 35S::PgMYB47-GFP, 35S::PgMYB142-GFP, and 35S::PgMYB151-GFP colocalization with DAPI-stained nuclei in *N. benthamiana* leaf epidermal cells. These results indicate that PgMYB47, PgMYB142, and PgMYB151 are all localized in the nucleus in *N. benthamiana* leaf epidermal cells, exhibiting the subcellular characteristics expected of transcription factors and suggesting that they may be involved in transcriptional regulation. However, because these localization results were obtained using a heterologous transient expression system, they have inherent limitations. 

## 4. Discussion

MYB transcription factors constitute one of the largest and most functionally diverse families of transcriptional regulators in plants, playing crucial roles in growth, development, and stress responses [1]. Although they have been extensively studied in species such as *Arabidopsis thaliana* and *Oryza sativa* [1,22], their characterization in many medicinal plants remains limited. In this study, a comprehensive genome-wide analysis was conducted, and 170 MYB members were identified in the medicinal plant *P*. *grandiflorus*. A distinct feature of the *PgMYB* family is the predominance of the 1R-MYB subfamily (52.9%), which contrasts with the typical dominance of the 2R-MYB subfamily in most plants like *Arabidopsis* and rice [1,22]. The observed predominance of 1R-MYBs (52.9%) was derived from a stringent identification pipeline that included independent HMMER and BLASTP searches followed by manual curation. This suggests that the high proportion of 1R-MYB is unlikely to be an artifact of search bias or annotation errors, although experimental validation of representative members is still warranted. Nonetheless, the 2R-MYB subfamily, though slightly less abundant (44.1%), remains highly significant, especially given its well-established and conserved role in regulating secondary metabolism across diverse plant species [23]. Although this composition could reflect genuine lineage-specific expansion, it may also be influenced by genome annotation quality or the inherent divergence of 1R-MYB sequences. Therefore, experimental validation of representative 1R-MYB members is required before drawing evolutionary conclusions.

Phylogenetic analysis classified the 170 *PgMYB* genes into 26 subgroups, providing evolutionary insights and aided in functional prediction. The clustering of *PgMYB* members with functionally characterized *AtMYBs* suggests potential functional conservation. For instance, *PgMYB151* groups with the flavonol-regulating clade (*AtMYB11/12/111*) in subgroup P17 (S7) [23], and *PgMYB142* clusters with *AtMYB123* (*TT2*), a known regulator of proanthocyanidin biosynthesis, suggesting that it may have a related function, but this requires experimental testing [24]. This finding implies that core regulatory modules for flavonoid biosynthesis are evolutionarily conserved. Gene structure and conserved motif analyses further supported functional relatedness within subgroups as well as differentiation between them, reflecting the family’s functional diversification [25]. Notably, the previously reported *PlgMYBR1* also belongs to the R2R3-MYB subfamily but acts as a transcriptional repressor [14], suggesting that both positive and negative MYB regulators exist in *P. grandiflorus* for fine-tuning flavonoid/anthocyanin accumulation.

Analyses of gene distribution and evolution revealed that the 170 *PgMYB* genes are unevenly distributed across the nine chromosomes, with a tendency to form tandem clusters at chromosomal ends—a pattern observed in species such as pepper and often attributed to tandem duplication events [26,27]. Furthermore, the identification of 36 syntenic blocks within the *P. grandiflorus* genome and 192 syntenic regions with *Arabidopsis* indicates that both tandem and segmental duplications, the latter under strong purifying selection, have been major drivers of the expansion of the *PgMYB* family [28]. The diversity in protein physicochemical properties and the nuclear localization predicted for 90% of PgMYB proteins, consistent with their role as transcription factors [29], underscores the family’s functional versatility, which likely extends beyond metabolism to development and stress responses.

Promoter cis-element analysis identified 19 types of elements, with ABA- and MeJA-responsive elements being the most abundant, suggesting the *PgMYB* family is widely involved in abiotic stress and exogenous hormone-mediated adaptive responses [30]. This was experimentally supported by RT-qPCR, which showed that selected *PgMYB* genes exhibited distinct response patterns to various hormones. Notably, *PgMYB47* was significantly upregulated by all four hormones tested, with the strongest induction by MeJA—a finding consistent with the observation that its promoter contains the highest number of MeJA-responsive elements. This highlights the close link between a transcription factor’s regulatory function and the cis-element composition of its promoter [27]. The expression patterns under MeJA treatment validated our transcriptome data and confirmed that the *PgMYB* family widely participates in MeJA-induced responses.

It is essential to emphasize that the following interpretations are based entirely on predicted interactions and correlations; they are intended only to generate testable hypotheses and do not constitute evidence of direct regulation. Building on this, phylogenetic, expression correlation, and predicted PPI network analyses were integrated to pinpoint key regulators of flavonoid biosynthesis. *PgMYB47* emerged as the most promising candidate. It showed the highest positive correlation with the expression of key structural genes (*C4H*, *4CL*, *CHI*, *DFR*) and was predicted to interact with their protein products (*C4H2*, *4CL1*, *CHS1*, *DFR1*). Coupled with its dramatic induction by MeJA and abundant MeJA-responsive promoter elements, *PgMYB47* therefore emerges as a strong hypothetical candidate that could play a positive role in MeJA-mediated flavonoid biosynthesis; however, functional validation (e.g., by overexpression or gene silencing) is required to confirm such a role. This hypothesis is supported by its dramatic induction by MeJA, its high expression correlation with several flavonoid structural genes, and the predicted interactions. Nevertheless, direct evidence, such as promoter binding or genetic complementation, is needed to establish its regulatory function. This role is analogous to central MYB switches in other medicinal plants, such as *GuMYBv6* in *Glycyrrhiza uralensis* (licorice) for isoflavonoid biosynthesis [31] and *GmMYB* in *Astragalus membranaceus* (milkvetch) for flavonoid accumulation [32]. In contrast to the negative regulatory role of *PlgMYBR1* [14], the candidate *PgMYB47* identified in this study exhibits expression patterns consistent with a positive activator under MeJA treatment, highlighting the functional diversity of the R2R3-MYB family in *P. grandiflorus*. The functional implications of *PgMYB142* (based on phylogenetic clustering) and *PgMYB151* (based on protein interaction despite weak expression correlation) in flavonoid pathways also warrant further investigation. The PPI network presented in this study is entirely predicted based on *Arabidopsis* homologs and does not represent experimentally validated interactions in *P. grandiflorus*. Moreover, interactions may occur only in specific cell types (e.g., root cortex vs. vascular bundle) and under specific physiological conditions. Therefore, future studies using cell-type-specific approaches (e.g., protoplast-based co-IP, in situ proximity ligation assays, or single-cell transcriptomics combined with PPI prediction) are required to determine whether and where these predicted interactions occur in native *P. grandiflorus* tissues.

Although PgMYB47, PgMYB142, and PgMYB151 were localized in the nucleus in this assay, their actual subcellular distribution in native *P. grandiflorus* tissues (e.g., root cortex or vascular bundles) remains to be determined. It should be noted that the subcellular localization experiments were performed transiently in *N. benthamiana* leaf epidermal cells, an artificial heterologous system. Protein localization patterns can be influenced by cell type, developmental stage, and endogenous cellular environment [33]. Therefore, future studies using *P. grandiflorus* protoplasts or stable transgenic lines expressing the native promoters are required to validate their real localization [34].

Based on these correlative and predictive findings, a hypothetical working model for MeJA-induced flavonoid biosynthesis can be proposed. In this model, MeJA perception would trigger a signaling cascade that could lead to the transcriptional activation of candidate MYB regulators such as PgMYB47; the PgMYB47 protein, once localized to the nucleus, might then activate the transcription of flavonoid biosynthetic genes (*PAL*, *C4H*, *4CL*, *CHS*, *DFR*, etc.), potentially driving metabolite flux. It must be emphasized that this model is strictly hypothesis-generating and requires direct experimental testing. This model aligns with established jasmonate signaling pathways but requires direct validation in *P. grandiflorus*, for instance, through chromatin immunoprecipitation and promoter-binding assays for PgMYB47. However, a full hormetic window was not established, the 12 h time point may capture adaptive/secondary responses rather than primary transcriptional events. Therefore, future time-course experiments including earlier time points (1–6 h) and multiple concentrations are needed.

It should be noted that all transcriptomic and RT-qPCR analyses were performed on whole-root homogenates, since gene expression and protein interactions are highly cell-type-specific [35]. For example, flavonoid biosynthesis and its transcriptional regulation may occur predominantly in specific root cell types such as the cortex, endodermis, or pericycle, rather than uniformly across all cells [36]. Therefore, the expression patterns and predicted interactions reported here represent averaged signals that may obscure cell-type-specific dynamics. Future studies using spatial transcriptomics, single-cell RNA-sequencing, or in situ hybridization will be necessary to resolve the cell-type-specific expression of *PgMYB* genes and their potential interactions with flavonoid biosynthetic enzymes in native *P. grandiflorus* roots.

It is worth noting that direct flavonoid metabolite measurements were not performed in the hormone-treated root samples in this study. Although *PgMYB47*, *PgMYB142*, and *PgMYB151* have been nominated as candidate regulators based on their expression patterns, predicted interactions, and phylogenetic relationships, their expression changes cannot be directly linked to actual flavonoid accumulation. Flavonoid levels may be influenced by post-transcriptional regulation, substrate availability, or other pathway-specific factors that our transcriptome data do not capture. Therefore, the study should be viewed as a candidate gene discovery effort rather than a functional validation study. Future work integrating metabolomics (e.g., LC-MS quantification of flavonoid compounds) with genetic perturbation (e.g., CRISPR-Cas9 or overexpression in *P. grandiflorus*) is required to establish the precise regulatory functions of these *PgMYB* genes in flavonoid biosynthesis.

## 5. Conclusions

In this study, 170 MYB transcription factor family members were identified from the *P. grandiflorus* genome, with the 1R-MYB and 2R-MYB subfamilies being dominant (52.9% and 44.1%, respectively). Numerous syntenic regions exist between *P. grandiflorus* and *Arabidopsis*, along with similar gene structures among *PgMYB* members, indicate that this family is evolutionarily conserved. Promoter cis-element analysis revealed that the upstream regions of *PgMYB* genes are enriched in elements related to hormone response, growth and development, and stress response. The ABA response element was the most widely distributed (72.9%), followed by the MeJA response element. Expression analysis indicated that the *PgMYB* gene family widely responds to MeJA treatment, with *PgMYB47* and *PgMYB142* showing the most significant responses, and RT-qPCR results were consistent with the transcriptome data. PPI network analysis predicted that ten PgMYB proteins interact with eight flavonoid synthesis-related proteins. Based on these findings, *PgMYB47*, *PgMYB142*, and *PgMYB151* are nominated as candidate regulators for future functional studies on flavonoid biosynthesis in *P. grandiflorus*. Notably, a previous study identified *PlgMYBR1* as a negative regulator of anthocyanin (a flavonoid subclass) biosynthesis in *P. grandiflorus* [14], indicating that both positive and negative MYB regulators exist in this species. However, direct flavonoid metabolite measurements were not performed in this study, and the proposed regulatory functions require experimental validation (e.g., transactivation assays, genetic perturbation, or direct binding tests). This study provides a comprehensive resource and a foundation for further functional characterization of the *PgMYB* family in secondary metabolism.

## Figures and Tables

**Table 1:** Primers of *PgMYB* genes for RT-qPCR.

Gene Name	Forward Primer (5′ → 3′)	Reverse Primer (5′ → 3′)
*PgMYB14*	CACCATTGCCCGTTTGCTT	CCACCGTCCACTCTCTCCC
*PgMYB15*	ACAGAGGCTCGTTGATAAA	GCGTAGAGTTCCAATGGTT
*PgMYB47*	TCATGCTAAGTGGGGAAAC	TGGGAAAAGGACCTGTAAA
*PgMYB62*	TTGGAGAACTCGTGTGCAG	ATTTGTCGTAGCGGATGGT
*PgMYB99*	TGGTGTGGATTCGTGTGAG	AGACCTGTTGGTTTGGCTG
*PgMYB111*	GCTTCCGCTAAGAAACCAA	GGGCCGTATCTCTCAACAA
*PgMYB142*	CTTGATGTTGATTTGTGGG	ATGAAGAAGAAGTTGCCGT
*PgMYB150*	ATGCTGTGAGAAGATGGGG	TTAATGTCGGGTCGGAGAT
*PgMYB167*	ACAATAACCAAACAACGGG	TCTATGTCAAGAGCCTGCC
*β-actin*	CCGTGGAGCCAAGGGTTG	GGAGCACCCAAGCTTGCG

## Data Availability

The processed datasets generated during this study have been deposited in the Figshare public repository. Specifically, the datasets include: (1) the Ks/Ka values for PgMYB gene pairs; (2) the expression levels (FPKM) of *PgMYB* gene members; and (3) the physicochemical properties of PgMYB proteins. These data are available at https://figshare.com/s/67b1a948758b16661bbf, accessed on 15 April 2025. All other relevant data are included in the manuscript and its Supplementary Files.

## Supplementary Materials

The following supporting information can be downloaded at: https://www.mdpi.com/article/10.3390/genes17060638/s1. Table S1. Physicochemical properties and subcellular localization of MYB protein in *P. grandifloras*, Table S2. Ka/Ks analysis of MYB gene in *P. grandiflorus* Figure S1. Maximum Likelihood (ML) phylogenetic tree of MYB gene family members from *Platycodon grandiflorus* and *Arabidopsis thaliana*. Subgroups P1–P26, defined by clustering with *Arabidopsis* MYBs of known subfamilies. The scale bar represents the number of substitutions per site. This ML tree confirms the topology of the NJ tree presented in Figure 1 of the main manuscript.
