# Supplementary material for: Genome-Wide Identification and Characterization of the MYB Transcription Factor Family in Platycodon grandiflorus and Its Potential Involvement in Flavonoid Biosynthesis Regulation
Source: Genes (Basel). 2026 May 30;17(6):638. doi: 10.3390/genes17060638 (PMC13298726; doi:10.3390/genes17060638)
Supplement: Supplementary file 1 [file genes-17-00638-s001.zip › genes-4321217-supplementary.pdf]

Table S1. Physicochemical properties and subcellular localization of MYB protein in *P. grandiflorus*

Gene name	Sequence ID	Number of Amino Acid	Molecular Weight	Theoretical pI	Instability Index	Aliphatic Index	Grand Average of Hydropathicity	Subcellular localization
PGRA_12899	PgMYB86	259	29948.29	5.12	52.35	69.73	-0.77	Nuclear
PGRA_11893	PgMYB87	361	40914.68	5.34	53.73	75.07	-0.653	Nuclear
PGRA_10498	PgMYB88	275	31285.06	8.94	42.09	64.22	-0.882	Nuclear
PGRA_10172	PgMYB89	425	48237.76	6.38	56.92	68.09	-0.823	Nuclear
PGRA_13473	PgMYB90	83	9401.39	4.86	50.06	66.99	-0.81	Chloroplast
PGRA_10672	PgMYB91	94	10489.64	5.8	42.21	63.3	-0.846	Chloroplast
PGRA_12679	PgMYB92	563	65045.32	8.92	65.41	67.82	-1.021	Cytoplasmic
PGRA_12402	PgMYB93	643	73285.98	8.19	61.84	65.74	-0.991	Nuclear
PGRA_10904	PgMYB94	298	33678.37	5.53	53.6	69.97	-0.733	Nuclear
PGRA_13462	PgMYB95	79	9084.07	9.45	82.21	61.77	-1.004	Nuclear
PGRA_12946	PgMYB96	251	28484.17	8.29	31.41	75.82	-0.68	Nuclear
PGRA_11462	PgMYB97	349	39657.46	6.4	47.66	64.27	-0.74	Nuclear
PGRA_11252	PgMYB98	138	15388.72	9.65	40.58	79.93	-0.612	Nuclear
PGRA_13438	PgMYB99	390	42355.53	9.2	60.13	65.74	-0.57	Nuclear
PGRA_11631	PgMYB100	387	43116.64	5.94	61.45	71.06	-0.724	Nuclear
PGRA_10862	PgMYB101	241	28118.59	6.25	54.15	62.24	-0.869	Nuclear
PGRA_12839	PgMYB102	289	33223.24	7.09	56.81	66.12	-0.945	Nuclear
PGRA_10054	PgMYB103	391	42602.31	6.23	53.49	64.86	-0.67	Nuclear
PGRA_11758	PgMYB104	350	38691.8	5.88	50.24	61.6	-0.722	Nuclear
PGRA_11530	PgMYB105	336	37659.32	6.52	51.06	68.21	-0.665	Nuclear
PGRA_11816	PgMYB106	698	77457.29	9.26	62.01	73.44	-0.561	Nuclear
PGRA_12832	PgMYB107	410	45155.12	6.68	44.53	73.05	-0.528	Nuclear
PGRA_12859	PgMYB108	279	32243.02	8.61	64.34	55.56	-0.831	Nuclear

Gene name	Sequence ID	Number of Amino Acid	Molecular Weight	Theoretical pI	Instability Index	Aliphatic Index	Grand Average of Hydropathicity	Subcellular localization
PGRA_10924	PgMYB109	364	41053.44	8.72	46.31	64.53	-0.958	Nuclear
PGRA_10928	PgMYB110	313	34349.79	9.87	55.63	62.62	-0.679	Nuclear
PGRA_13419	PgMYB111	208	23455.08	9.74	54.22	58.17	-0.951	Nuclear
PGRA_12535	PgMYB112	292	33022.01	5.53	56.63	69.18	-0.628	Nuclear
PGRA_11157	PgMYB113	281	31657.45	5.4	56.07	67.33	-0.679	Nuclear
PGRA_12663	PgMYB114	328	36202.73	7.73	46.57	66.92	-0.622	Nuclear
PGRA_12838	PgMYB115	330	37443.69	4.98	55.88	68.85	-0.628	Nuclear
PGRA_21818	PgMYB1	621	69020.79	6.26	45.95	78.13	-0.605	Nuclear
PGRA_20451	PgMYB2	276	30716.07	8.08	41.28	83.8	-0.518	Cytoplasmic
PGRA_20353	PgMYB3	1215	134964.37	7.05	44.04	80.35	-0.407	Nuclear
PGRA_21876	PgMYB4	346	39596.14	5.97	52.13	58.64	-0.812	Nuclear
PGRA_19674	PgMYB5	453	51490.08	4.97	55.6	61.74	-0.873	Nuclear
PGRA_18680	PgMYB6	314	34134.37	8.9	56.92	73.25	-0.43	Nuclear
PGRA_19551	PgMYB7	332	37096.81	5.73	46.62	72.32	-0.561	Nuclear
PGRA_20223	PgMYB8	341	37808.12	6.26	52.82	66.69	-0.697	Nuclear
PGRA_19536	PgMYB9	503	57295	4.45	60	61.55	-0.709	Peroxisomal
PGRA_21112	PgMYB10	298	32692.49	8.59	54.26	55.6	-0.761	Nuclear
PGRA_21143	PgMYB11	99	11304.69	9.16	68.16	61.11	-0.86	Nuclear
PGRA_19272	PgMYB12	98	11086.31	6.55	50.91	69.69	-0.855	Nuclear
PGRA_18720	PgMYB13	360	39582.66	6.16	46.49	69.64	-0.669	Nuclear
PGRA_20361	PgMYB14	233	25907.72	8.6	49.9	61.16	-0.826	Nuclear
PGRA_19664	PgMYB15	192	21733.62	8.98	55.96	75.21	-0.658	Nuclear
PGRA_21651	PgMYB16	335	37921.78	7.88	56.82	68.69	-0.753	Nuclear
PGRA_20630	PgMYB17	463	47774.81	9.29	56.47	80.13	-0.376	Nuclear

Gene name	Sequence ID	Number of Amino Acid	Molecular Weight	Theoretical pI	Instability Index	Aliphatic Index	Grand Average of Hydropathicity	Subcellular localization
PGRA_21176	PgMYB18	271	29967.44	6.54	33.27	74.13	-0.754	Nuclear
PGRA_20366	PgMYB19	334	40119.63	9.49	59.53	50.54	-1.442	Peroxisomal
PGRA_20912	PgMYB20	333	37023.6	5.88	44.4	80.21	-0.637	Nuclear
PGRA_20205	PgMYB21	310	34766.44	9.14	42.98	79.29	-0.543	Nuclear
PGRA_19817	PgMYB22	506	55912.03	4.42	38.18	65.08	-0.626	Nuclear
PGRA_19949	PgMYB23	289	30852.08	6.37	44.21	62.08	-0.607	Nuclear
PGRA_19686	PgMYB24	390	42179.74	8.93	63.67	51.56	-0.852	Nuclear
PGRA_20116	PgMYB25	301	34297.93	6.2	64.65	59.63	-0.983	Nuclear
PGRA_20594	PgMYB26	158	18165.64	9.89	39.02	68.61	-0.961	Nuclear
PGRA_19634	PgMYB27	321	36041.47	7.02	47.33	71.34	-0.672	Nuclear
PGRA_07267	PgMYB116	521	55574.24	6.91	62.03	60.98	-0.705	Nuclear
PGRA_05112	PgMYB117	356	39437.52	5.11	54.11	72.05	-0.74	Nuclear
PGRA_05728	PgMYB118	280	30886.87	6.5	45.28	74.57	-0.546	Nuclear
PGRA_06034	PgMYB119	78	8671.73	9.05	57.52	62.56	-0.668	Cytoplasmic
PGRA_06105	PgMYB120	983	107368.44	4.8	52.57	70.79	-0.625	Nuclear
PGRA_05560	PgMYB121	426	48637.03	7.22	61.33	66.13	-0.793	Nuclear
PGRA_05766	PgMYB122	500	56487.73	5.55	47.45	74.12	-0.68	Nuclear
PGRA_05512	PgMYB123	103	11562.99	6.57	39.18	67.18	-0.78	Nuclear
PGRA_04623	PgMYB124	468	51166.81	5.69	49.73	68.8	-0.567	Nuclear
PGRA_06379	PgMYB125	301	33165.23	6.62	51.83	73.22	-0.625	Nuclear
PGRA_04860	PgMYB126	301	32860.77	9.61	43.38	79.97	-0.449	Nuclear
PGRA_06963	PgMYB127	535	59234.5	5.99	59.81	73.79	-0.573	Nuclear
PGRA_06711	PgMYB128	775	85956.67	6.53	42.73	72.76	-0.551	Peroxisomal
PGRA_04808	PgMYB129	303	33814.68	7.63	50.38	61.16	-0.739	Nuclear

Gene name	Sequence ID	Number of Amino Acid	Molecular Weight	Theoretical pI	Instability Index	Aliphatic Index	Grand Average of Hydropathicity	Subcellular localization
PGRA_06508	PgMYB130	307	34426.72	6.51	59.26	69.87	-0.681	Nuclear
PGRA_06251	PgMYB131	388	44855.69	9.18	61.12	65.05	-0.886	Nuclear
PGRA_07134	PgMYB132	1043	115519.46	5.25	53.27	72.79	-0.723	Nuclear
PGRA_05454	PgMYB133	400	45387.57	6.43	62.86	64.82	-0.793	Nuclear
PGRA_05592	PgMYB134	326	37235.34	6.12	38.53	68.77	-0.873	Nuclear
PGRA_05632	PgMYB135	394	43835.26	6.54	39.6	76.24	-0.656	Nuclear
PGRA_08899	PgMYB61	499	55255.17	5.53	48.65	75.83	-0.511	Chloroplast
PGRA_07655	PgMYB62	272	31400.5	6.63	57	78.16	-0.722	Nuclear
PGRA_09723	PgMYB63	1163	131561.82	8.15	53.9	68.11	-0.82	Nuclear
PGRA_07342	PgMYB64	272	31785.42	7.81	62.18	61.36	-0.975	Nuclear
PGRA_09250	PgMYB65	245	27983.77	6.66	52.9	72.86	-0.684	Nuclear
PGRA_08818	PgMYB66	262	29943.34	6.01	39.02	81.03	-0.469	Nuclear
PGRA_09679	PgMYB67	290	31600.19	9.78	63.82	65.62	-0.664	Nuclear
PGRA_08947	PgMYB68	467	52806.98	5.43	55.42	76.02	-0.687	Nuclear
PGRA_09287	PgMYB69	690	77162.68	4.75	61.62	58.55	-0.96	Nuclear
PGRA_07563	PgMYB70	361	40390.39	6.31	57.6	67.01	-0.581	Nuclear
PGRA_08190	PgMYB71	794	86079.75	5.55	47.52	75.19	-0.495	Nuclear
PGRA_08850	PgMYB72	328	37340.28	9.63	54.14	58.57	-0.963	Nuclear
PGRA_08536	PgMYB73	305	34337.71	6.15	48.85	79.02	-0.548	Nuclear
PGRA_09906	PgMYB74	370	41536.83	8.82	56.57	60.65	-0.834	Nuclear
PGRA_09635	PgMYB75	320	36460.61	5.83	40.23	84.06	-0.49	Nuclear
PGRA_07647	PgMYB76	333	37467.28	6.53	47.53	82.94	-0.521	Nuclear
PGRA_08168	PgMYB77	561	62588.82	5.86	50.44	73.65	-0.546	Cytoplasmic
PGRA_08203	PgMYB78	322	35605.12	7.77	64.97	71.77	-0.521	Nuclear

Gene name	Sequence ID	Number of Amino Acid	Molecular Weight	Theoretical pI	Instability Index	Aliphatic Index	Grand Average of Hydropathicity	Subcellular localization
PGRA_08425	PgMYB79	115	12526.47	8.89	52.24	80.7	-0.213	Nuclear
PGRA_09450	PgMYB80	376	41357.49	9.11	32.95	45.11	-1.028	Nuclear
PGRA_09854	PgMYB81	367	39442.76	6.92	56.7	59.81	-0.613	Nuclear
PGRA_09825	PgMYB82	263	29783.07	4.82	47.83	68.97	-0.784	Nuclear
PGRA_09623	PgMYB83	541	60214.5	7.46	58.65	65.43	-0.654	Nuclear
PGRA_09637	PgMYB84	302	34350.46	9.43	44.59	64.9	-0.817	Nuclear
PGRA_09034	PgMYB85	414	46033.72	6.09	48.35	76.62	-0.581	Nuclear
PGRA_04465	PgMYB45	339	38291.7	5.56	49.23	71.65	-0.657	Nuclear
PGRA_04231	PgMYB46	254	29486.84	6.41	61.85	59.13	-0.966	Nuclear
PGRA_03145	PgMYB47	182	21036.42	6.3	53.98	60.11	-0.879	Nuclear
PGRA_04033	PgMYB48	258	29523.69	5.38	62.12	67.33	-0.859	Nuclear
PGRA_02556	PgMYB49	268	30963.67	4.96	49.3	76.38	-0.847	Nuclear
PGRA_04311	PgMYB50	121	13906.34	9.61	65.5	85.37	-0.463	Nuclear
PGRA_04397	PgMYB51	306	33539.64	8.37	58.97	73.89	-0.703	Nuclear
PGRA_04278	PgMYB52	377	42954.29	6.91	57.9	61.03	-1.049	Nuclear
PGRA_02791	PgMYB53	290	33127.89	7.76	52.24	55.86	-0.886	Nuclear
PGRA_02246	PgMYB54	432	47634.68	9.09	44.14	74.77	-0.524	Nuclear
PGRA_03749	PgMYB55	538	59106.51	8.64	60.29	63.66	-0.762	Nuclear
PGRA_02766	PgMYB56	279	31046.66	7.71	52.88	80	-0.174	Chloroplast
PGRA_02301	PgMYB57	1172	130163.39	9.28	50.27	76.02	-0.63	Chloroplast
PGRA_03841	PgMYB58	489	55518.77	5.58	45.68	84.07	-0.616	Nuclear
PGRA_03399	PgMYB59	381	42553.24	6.89	44.49	63.49	-0.753	Nuclear
PGRA_02733	PgMYB60	433	47251.36	5.97	61.28	72.7	-0.476	Nuclear
PGRA_18033	PgMYB136	323	36479.79	5.52	56.67	65.2	-0.644	Nuclear

Gene name	Sequence ID	Number of Amino Acid	Molecular Weight	Theoretical pI	Instability Index	Aliphatic Index	Grand Average of Hydropathicity	Subcellular localization
PGRA_18270	PgMYB137	692	76407.59	8.98	50.48	75.79	-0.547	Chloroplast
PGRA_16281	PgMYB138	194	22183.52	5.07	54.1	48.76	-1.004	Nuclear
PGRA_16403	PgMYB139	999	114657.61	8.81	38.26	62.52	-0.894	Nuclear
PGRA_17800	PgMYB140	363	40996.35	7.11	55.51	59.7	-0.852	Nuclear
PGRA_18301	PgMYB141	529	57945.26	5.22	57.06	70.26	-0.57	Nuclear
PGRA_18135	PgMYB142	288	32922.89	7.08	49.31	62.33	-0.894	Nuclear
PGRA_16141	PgMYB143	281	31240.44	9.15	45.38	71.21	-0.703	Nuclear
PGRA_17863	PgMYB144	309	34864.1	9.2	49.97	55.89	-0.845	Nuclear
PGRA_17732	PgMYB145	328	37627.36	8.99	48.15	67.41	-0.786	Nuclear
PGRA_16890	PgMYB146	489	55697.9	5.45	45.76	85.07	-0.645	Nuclear
PGRA_17584	PgMYB147	245	26572.8	8.95	45.79	75.71	-0.669	Nuclear
PGRA_18067	PgMYB148	1948	210376.17	7.12	57.03	64.07	-0.726	Nuclear
PGRA_17467	PgMYB149	493	53718.99	5.62	59.83	71.42	-0.615	Nuclear
PGRA_17933	PgMYB150	283	32635.34	5.42	46.6	62.72	-0.881	Nuclear
PGRA_16146	PgMYB151	400	45255.45	4.83	55.36	70.67	-0.749	Nuclear
PGRA_13525	PgMYB152	1036	114471.78	5.07	59.73	64.93	-0.659	Nuclear
PGRA_14086	PgMYB153	233	25791.1	8.22	49.2	70.34	-0.515	Nuclear
PGRA_13612	PgMYB154	272	30078.66	8.84	49.57	64.85	-0.556	Chloroplast
PGRA_15086	PgMYB155	1222	136386.58	9.19	52.33	69.77	-0.745	Nuclear
PGRA_15058	PgMYB156	679	74897.9	5.95	47.58	77.64	-0.544	Nuclear
PGRA_14339	PgMYB157	286	31837.81	6.38	56.65	75.63	-0.729	Nuclear
PGRA_14869	PgMYB158	689	75272.37	8.68	51.89	80.96	-0.339	Nuclear
PGRA_14902	PgMYB159	544	61325.35	6.57	57.34	70.68	-0.729	Nuclear
PGRA_13953	PgMYB160	321	36611.46	5.45	78.61	54.42	-0.931	Nuclear

Gene name	Sequence ID	Number of Amino Acid	Molecular Weight	Theoretical pI	Instability Index	Aliphatic Index	Grand Average of Hydropathicity	Subcellular localization
PGRA_13717	PgMYB161	332	36229.03	5.85	47.19	61.78	-0.654	Nuclear
PGRA_14396	PgMYB162	483	53639.62	6.07	63.49	52.26	-0.811	Nuclear
PGRA_14438	PgMYB163	434	48034.19	7.25	57.05	60.18	-0.808	Nuclear
PGRA_13989	PgMYB164	309	35104.19	4.82	68.68	78.28	-0.695	Nuclear
PGRA_14011	PgMYB165	338	37505.5	5.89	55.05	78.22	-0.504	Nuclear
PGRA_14913	PgMYB166	674	75184.29	6.03	40.28	73.47	-0.594	Nuclear
PGRA_01713	PgMYB28	340	38007.13	6.06	48.93	60.35	-0.814	Nuclear
PGRA_00798	PgMYB29	311	33642.63	9.36	55.21	69.68	-0.489	Chloroplast
PGRA_01689	PgMYB30	274	30711.31	7.1	49.97	63.03	-0.661	Nuclear
PGRA_00479	PgMYB31	247	26975.54	8.96	52.69	77.94	-0.604	Nuclear
PGRA_00595	PgMYB32	143	15532.19	6.36	60.86	68.32	-0.595	Nuclear
PGRA_01643	PgMYB33	171	19826.46	9.18	44.48	67.25	-0.847	Nuclear
PGRA_01918	PgMYB34	94	11112.46	5.43	58.84	72.66	-0.533	Nuclear
PGRA_00448	PgMYB35	171	19784.4	9.18	44.48	64.97	-0.88	Nuclear
PGRA_01994	PgMYB36	302	32803.91	6.19	52.49	80.5	-0.46	Nuclear
PGRA_01768	PgMYB37	225	26304.77	6.22	66.9	71.07	-0.761	Nuclear
PGRA_01006	PgMYB38	404	44013.34	7.32	54.81	58.27	-0.905	Nuclear
PGRA_00110	PgMYB39	671	72879.52	5.9	40.48	73.35	-0.503	Nuclear
PGRA_01238	PgMYB40	296	33294.26	8.56	33.7	84.93	-0.485	Cytoplasmic
PGRA_00472	PgMYB41	310	34596.38	5.37	49.43	60.77	-0.813	Nuclear
PGRA_01567	PgMYB42	269	30525.24	5.46	53.47	72.53	-0.777	Cytoplasmic
PGRA_00173	PgMYB43	440	49079.83	5.3	56.57	68.95	-0.74	Nuclear
PGRA_00368	PgMYB44	257	29258.39	5.38	50.7	66.34	-0.779	Nuclear
PGRA_15275	PgMYB167	771	84362.26	6.17	52.19	63.26	-0.731	Nuclear

Gene name	Sequence ID	Number of Amino Acid	Molecular Weight	Theoretical pI	Instability Index	Aliphatic Index	Grand Average of Hydropathicity	Subcellular localization
PGRA_15861	PgMYB168	307	34385.14	8.69	62.17	68.96	-0.62	Nuclear
PGRA_15356	PgMYB169	1142	128432.85	6.98	42.55	74.28	-0.606	Nuclear
PGRA_15589	PgMYB170	619	70133.67	5.68	56.28	72.39	-0.586	Nuclear

Table S2. Ka/Ks analysis of MYB gene in *P. grandiflorus*

Seq_1	Seq_2	Ka	Ks	Ka_Ks	EffectiveLen	AverageS-sites	AverageN-sites	cN	cS	pN	pS
PGRA_04465	*PGRA_11893*	0.244219441	1.759376308	0.138810236	966	210	756	157.5833333	142.4166667	0.208443563	0.678174603
PGRA_21143	*PGRA_13462*	0.271893526	3.566807283	0.076228824	234	51.66666667	182.3333333	41.58333333	38.41666667	0.228062157	0.743548387
PGRA_21651	*PGRA_11462*	0.338744369	2.185765292	0.154977467	990	211	779	212.3333333	149.6666667	0.272571673	0.709320695
PGRA_19664	*PGRA_13438*	0.227472179	5.832715802	0.038999359	564	132.5	431.5	84.66666667	99.33333333	0.196214755	0.749685535
PGRA_20205	*PGRA_10054*	0.385021371	2.915107387	0.132077937	879	199.0833333	679.9166667	204.75	146.25	0.301139846	0.734616995
PGRA_20223	*PGRA_11758*	0.298200119	1.97225458	0.15119758	918	200.3333333	717.6666667	176.5833333	139.4166667	0.24605202	0.695923461
PGRA_19551	*PGRA_11530*	0.461652433	3.430626178	0.13456798	933	204.6666667	728.3333333	251.0833333	151.9166667	0.344736842	0.742263844
PGRA_11816	*PGRA_18270*	0.41674535	1.314404067	0.317060302	2031	475	1556	497.5	294.5	0.319730077	0.62
PGRA_02246	*PGRA_12832*	0.349546006	1.998059898	0.174942706	1074	248.4166667	825.5833333	230.6666667	173.3333333	0.279398405	0.697752432
PGRA_12859	*PGRA_17863*	0.314003024	1.781914279	0.176216683	822	177.25	644.75	165.4166667	120.5833333	0.25655939	0.680300893
PGRA_04278	*PGRA_10924*	0.425398415	1.557533972	0.273123041	1005	213.4166667	791.5833333	257	140	0.324665754	0.655993752
PGRA_11157	*PGRA_15861*	0.328696048	2.435907373	0.134937827	831	186.5833333	644.4166667	171.5	134.5	0.266132161	0.720857526
PGRA_20451	*PGRA_16141*	0.258849888	1.845623244	0.140250665	801	181.25	619.75	135.6666667	124.3333333	0.218905473	0.685977011
PGRA_19674	*PGRA_04033*	0.516779338	2.238002762	0.230910947	672	138.9166667	533.0833333	199.0833333	98.91666667	0.373456308	0.712057588
PGRA_19536	*PGRA_19817*	0.511822716	2.569758276	0.199171541	1260	275.1666667	984.8333333	365.3333333	199.6666667	0.370959553	0.725620836
PGRA_21143	*PGRA_04878*	0.341273979	1.99996812	0.170639709	243	55.16666667	187.8333333	51.5	38.5	0.274179237	0.697885196
PGRA_20116	*PGRA_07655*	0.400116541	1.48752294	0.268981762	795	167.75	627.25	194.5	108.5	0.310083699	0.646795827
PGRA_05766	*PGRA_14902*	0.23664632	1.551883336	0.152489762	1398	312.3333333	1085.666667	220.3333333	204.6666667	0.202947498	0.655282818
PGRA_04808	*PGRA_17800*	0.338256691	1.502874598	0.225073131	846	179.6666667	666.3333333	181.4166667	116.5833333	0.272261131	0.648886827
PGRA_02556	*PGRA_09250*	0.280900803	1.837060652	0.152907746	660	136.0833333	523.9166667	122.75	93.25	0.234292986	0.685241886
PGRA_08536	*PGRA_14011*	0.339245025	1.583454139	0.21424367	894	196.8333333	697.1666667	190.25	129.75	0.27289027	0.65918713
PGRA_09906	*PGRA_09637*	0.483560778	2.476103013	0.195291058	774	178	596	212.4166667	128.5833333	0.356403803	0.722378277
PGRA_09623	*PGRA_15356*	0.563818695	1.894913781	0.297543192	1425	320.75	1104.25	437.6666667	221.3333333	0.396347445	0.690049363
PGRA_02301	*PGRA_15086*	0.410751615	1.343819602	0.305659788	3408	794	2614	826.75	496.25	0.316277735	0.625

Seq_1	Seq_2	Ka	Ks	Ka_Ks	EffectiveLen	AverageS-sites	AverageN-sites	cN	cS	pN	pS
PGRA_00368	*PGRA_17933*	0.411630796	1.911218236	0.215376135	753	158.75	594.25	188.25	109.75	0.316785865	0.691338583
PGRA_01689	*PGRA_14086*	0.333811946	0.98887672	0.337566796	588	134.25	453.75	122.25	73.75	0.269421488	0.549348231

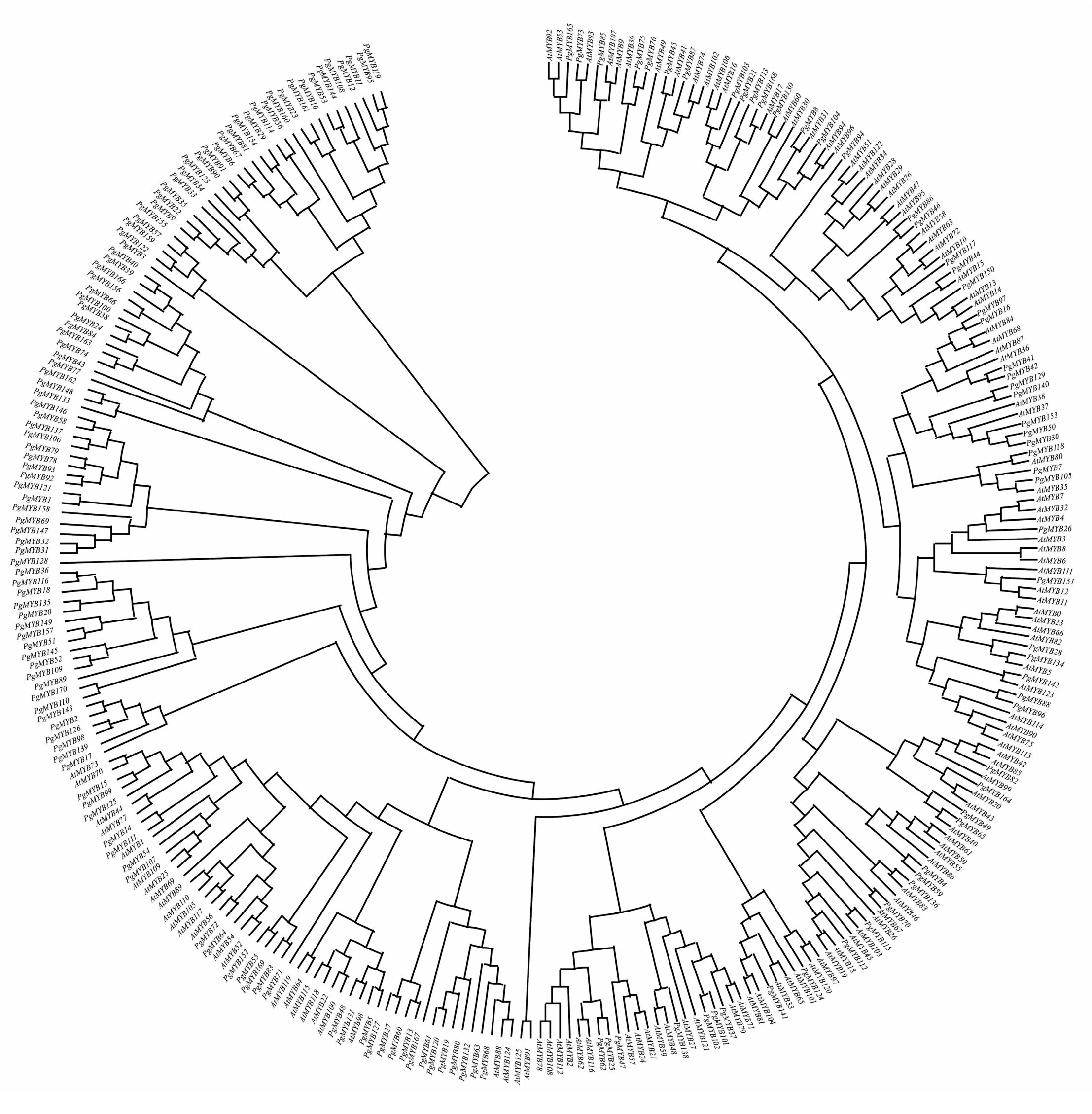

Supplementary Figure S1 Maximum Likelihood (ML) phylogenetic tree of MYB gene family members from *Platycodon grandiflorus* and *Arabidopsis thaliana*.

Subgroups P1–P26, defined by clustering with *Arabidopsis* MYBs of known subfamilies. The scale bar represents the number of substitutions per site. This ML tree confirms the topology of the NJ tree presented in Figure 1 of the main manuscript.

Supplementary method:

Construction of the Maximum Likelihood (ML) phylogenetic tree

Multiple sequence alignments of the MYB protein sequences were performed using MEGA11 (Version 11.0.13; Mega Software, Temple University, Philadelphia, PA, USA). The best-fit substitution model was selected based on the Bayesian Information Criterion (BIC). The Maximum Likelihood (ML) phylogenetic tree was constructed using the Jones-Taylor-Thornton (JTT) amino acid substitution model with uniform rates among sites. All positions containing gaps and missing data were retained (use all sites). The tree inference was conducted using the Nearest-Neighbor-Interchange (NNI) heuristic method, with the initial tree generated automatically via the NJ/BioNJ algorithm. To expedite the computation, 8 parallel threads were utilized. Branch support was assessed using 1000 bootstrap replicates.
